# Dibenzo[*b*,*e*]thiepin-11(6*H*)-one

**DOI:** 10.1107/S1600536810000796

**Published:** 2010-01-13

**Authors:** Jerry P. Jasinski, Q. N. M. Hakim Al-arique, Ray J. Butcher, H. S. Yathirajan, B. Narayana

**Affiliations:** a Department of Chemistry, Keene State College, 229 Main Street, Keene, NH 03435-2001, USA; b Department of Studies in Chemistry, University of Mysore, Manasagangotri, Mysore 570 006, India; c Department of Chemistry, Howard University, 525 College Street NW, Washington, DC 20059, USA; d Department of Studies in Chemistry, Mangalore University, Mangalagangotri 574 199, India

## Abstract

In the title compound, C_14_H_10_OS, the seven-membered thiepin ring adopts a distorted boat conformation with the dihedral angle between the mean planes of the two fused benzene rings being 56.5 (1)°.

## Related literature

For the biological and chiroptical properties of dibenzo[*c*,*e*]thiepine derivatives, see: Rajsner *et al.* (1969 ▶, 1971 ▶); Truce & Emrick (1956 ▶); Tomascovic *et al.* (2000 ▶). For spectral, structural and theoretical studies of eight related 6-aryl­idenedibenzo[*b*,*e*]thiepin-11-one-5,5-dioxides, see: Kolehmainen *et al.* (2007 ▶). For DFT calculations and the *GAUSSIAN03* program package, see: Schmidt & Polik (2007 ▶); Frisch *et al.* (2004 ▶). For a description of the Cambridge Structural Database, see: Allen (2002 ▶).
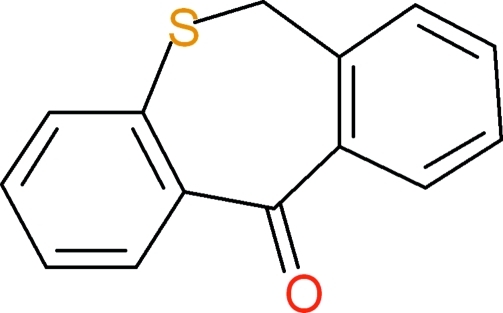


## Experimental

### 

#### Crystal data

C_14_H_10_OS
*M* *_r_* = 226.28
Orthorhombic, 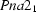

*a* = 14.6208 (11) Å
*b* = 4.3503 (3) Å
*c* = 16.9023 (13) Å
*V* = 1075.07 (14) Å^3^
*Z* = 4
Mo *K*α radiation
μ = 0.27 mm^−1^
*T* = 110 K
0.47 × 0.42 × 0.12 mm

#### Data collection

Oxford Diffraction Gemini R CCD diffractometer
Absorption correction: multi-scan (*CrysAlis RED*; Oxford Diffraction, 2007 ▶) *T*_min_ = 0.769, *T*_max_ = 1.000
2124 measured reflections
1322 independent reflections
1291 reflections with *I* > 2σ(*I*)
*R*_int_ = 0.022

#### Refinement

*R*[*F* ^2^ > 2σ(*F* ^2^)] = 0.039
*wR*(*F* ^2^) = 0.104
*S* = 1.06
1322 reflections
145 parameters
1 restraint
H-atom parameters constrained
Δρ_max_ = 0.42 e Å^−3^
Δρ_min_ = −0.35 e Å^−3^
Absolute structure: Flack (1983 ▶), 242 Friedel pairs
Flack parameter: −0.57 (13)

### 

Data collection: *CrysAlis PRO* (Oxford Diffraction, 2007 ▶); cell refinement: *CrysAlis PRO*; data reduction: *CrysAlis PRO*; program(s) used to solve structure: *SHELXS97* (Sheldrick, 2008 ▶); program(s) used to refine structure: *SHELXL97* (Sheldrick, 2008 ▶); molecular graphics: *SHELXTL* (Sheldrick, 2008 ▶); software used to prepare material for publication: *SHELXTL* and *WebMOPro* (Schmidt & Polik, 2007 ▶).

## Supplementary Material

Crystal structure: contains datablocks global, I. DOI: 10.1107/S1600536810000796/ci2983sup1.cif

Structure factors: contains datablocks I. DOI: 10.1107/S1600536810000796/ci2983Isup2.hkl

Additional supplementary materials:  crystallographic information; 3D view; checkCIF report

## Figures and Tables

**Fig. 1.**
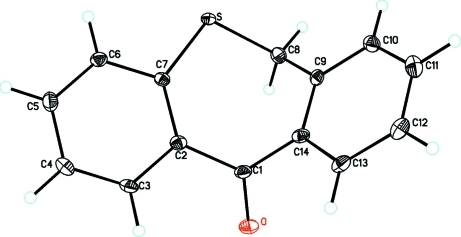
Molecular structure of the title compound, showing atom-labeling scheme and 50% probability displacement ellipsoids.

## supplementary crystallographic information

### Comment

The title compound is used as an intermediate for the synthesis of dosulepin, which is an antidepressant of the tricyclic family. Dosulepin works by preventing serotonin and noradrenaline from being reabsorbed in the brain. This helps prolong the mood lightening effect of any released noradrenaline and serotonin, thus relieving depression. The dibenzo[c,e]thiepine derivatives (Truce *et al.*, 1956) exhibit remarkable chiroptical properties (Tomascovic *et al.*, 2000). The dibenzo[b,e]thiepin-5,5-dioxide derivatives are known to possess antihistaminic and antiallergenic activities (Rajsner *et al.*, 1971). In addition, by aminoalkylation of 6,11-dihydrodibenzo[b,e]thiepin-5,5-dioxide and the corresponding 11-ketone, compounds with neurotropic and psychotropic activities have been reported (Rajsner *et al.*, 1969). In addition, the comparative NMR and IR spectral, X-ray structural and theoretical studies of eight 6-arylidenedibenzo[b,e]thiepin-11-one-5,5-dioxides have been reported (Kolehmainen *et al.*, 2007). In view of the importance of thiepines, this paper reports the crystal structure of the title compound.

The seven-membered thiepin ring adopts a distorted boat conformation with the dihedral angle between the mean planes of the two fused benzene rings measuring 56.5 (1)° (Fig. 1). This conformation is assisted by *sp*^3^ hybridization of atom C8 within the ring. The ketone oxygen atom (O) lies in an equatorial position from the ring on opposite sides of the C8 and S atoms [C3—C2—C1—O = 31.1 (4)° and C13—C14—C1—O = -50.7 (4)°]. Bond lengths and bond angles are all within expected ranges (Allen, 2002).

Following a geometry optimization density functional theory calculation (Schmidt & Polik, 2007), *in vacuo*, at the B3LYP 6–31-G(*d*) level with the GAUSSIAN03 program package (Frisch *et al.*, 2004) the angle between the mean planes of the two benzene rings becomes 46.2 (6)°, a decrease of 14.2 (5)°. The C3—C2—C1—O and C13—C14—C1—O torsion angles become 12.8 (2)°) and -36.9 (1)°, a decrease of 18.3 (2)° and 13.8 (3)°, respectively.

### Experimental

The title compound was obtained as a gift sample from *R. L*. Fine Chem, Bangalore, India. The compound was used without further purification. X-ray quality crystals (m.p. 347–349 K) were obtained by slow evaporation of a solution in methanol.

### Refinement

H atoms were placed in their calculated positions and then refined using the riding model with C–H = 0.95–0.99 Å, and with *U*_iso_(H) = 1.18–1.21*U*_eq_(C). The absolute structure could not be determined reliably due to the low Friedel pair coverage.

### Crystal data

**Table:**

C_14_H_10_OS	*F*(000) = 472
*M**_r_* = 226.28	*D*_x_ = 1.398 Mg m^−^^3^
Orthorhombic, *P**n**a*2_1_	Mo *K*α radiation, λ = 0.71073 Å
Hall symbol: P 2c -2n	Cell parameters from 1716 reflections
*a* = 14.6208 (11) Å	θ = 4.0–73.6°
*b* = 4.3503 (3) Å	µ = 0.27 mm^−^^1^
*c* = 16.9023 (13) Å	*T* = 110 K
*V* = 1075.07 (14) Å^3^	Plate, colorless
*Z* = 4	0.47 × 0.42 × 0.12 mm

### Data collection

**Table:**

Oxford Diffraction Gemini R CCD diffractometer	1322 independent reflections
Radiation source: fine-focus sealed tube	1291 reflections with *I* > 2σ(*I*)
graphite	*R*_int_ = 0.022
Detector resolution: 10.5081 pixels mm^-1^	θ_max_ = 26.2°, θ_min_ = 3.7°
φ and ω scans	*h* = −11→17
Absorption correction: multi-scan (*CrysAlis RED*; Oxford Diffraction, 2007)	*k* = −4→5
*T*_min_ = 0.769, *T*_max_ = 1.000	*l* = −8→20
2124 measured reflections	

### Refinement

**Table:**

Refinement on *F*^2^	Secondary atom site location: difference Fourier map
Least-squares matrix: full	Hydrogen site location: inferred from neighbouring sites
*R*[*F*^2^ > 2σ(*F*^2^)] = 0.039	H-atom parameters constrained
*wR*(*F*^2^) = 0.104	*w* = 1/[σ^2^(*F*_o_^2^) + (0.0678*P*)^2^ + 0.7978*P*] where *P* = (*F*_o_^2^ + 2*F*_c_^2^)/3
*S* = 1.06	(Δ/σ)_max_ = 0.001
1322 reflections	Δρ_max_ = 0.42 e Å^−^^3^
145 parameters	Δρ_min_ = −0.35 e Å^−^^3^
1 restraint	Absolute structure: Flack (1983), 242 Friedel pairs
Primary atom site location: structure-invariant direct methods	Flack parameter: −0.57 (13)

### Special details

**Table:**

Geometry. All e.s.d.'s (except the e.s.d. in the dihedral angle between two l.s. planes) are estimated using the full covariance matrix. The cell e.s.d.'s are taken into account individually in the estimation of e.s.d.'s in distances, angles and torsion angles; correlations between e.s.d.'s in cell parameters are only used when they are defined by crystal symmetry. An approximate (isotropic) treatment of cell e.s.d.'s is used for estimating e.s.d.'s involving l.s. planes.
Refinement. Refinement of *F*^2^ against ALL reflections. The weighted *R*-factor *wR* and goodness of fit *S* are based on *F*^2^, conventional *R*-factors *R* are based on *F*, with *F* set to zero for negative *F*^2^. The threshold expression of *F*^2^ > σ(*F*^2^) is used only for calculating *R*-factors(gt) *etc*. and is not relevant to the choice of reflections for refinement. *R*-factors based on *F*^2^ are statistically about twice as large as those based on *F*, and *R*- factors based on ALL data will be even larger.

### Fractional atomic coordinates and isotropic or equivalent isotropic displacement parameters (Å^2^)

**Table:**

	*x*	*y*	*z*	*U*_iso_*/*U*_eq_	
S	0.52142 (4)	−0.06772 (16)	0.24929 (6)	0.0178 (2)	
O	0.78147 (17)	−0.0883 (7)	0.09651 (19)	0.0321 (7)	
C1	0.7100 (2)	0.0006 (8)	0.1263 (2)	0.0200 (7)	
C2	0.7074 (2)	0.0905 (7)	0.2121 (2)	0.0180 (7)	
C3	0.7902 (2)	0.2143 (7)	0.2405 (2)	0.0227 (7)	
H3A	0.8423	0.2129	0.2070	0.027*	
C4	0.7980 (2)	0.3374 (9)	0.3154 (2)	0.0256 (8)	
H4A	0.8543	0.4237	0.3326	0.031*	
C5	0.7227 (2)	0.3339 (9)	0.3654 (2)	0.0244 (7)	
H5A	0.7276	0.4167	0.4173	0.029*	
C6	0.6408 (2)	0.2104 (7)	0.3401 (2)	0.0192 (7)	
H6A	0.5897	0.2100	0.3748	0.023*	
C7	0.6316 (2)	0.0853 (6)	0.26388 (19)	0.0161 (7)	
C8	0.5289 (2)	−0.2607 (8)	0.1555 (2)	0.0207 (7)	
H8A	0.5805	−0.4078	0.1569	0.025*	
H8B	0.4720	−0.3796	0.1466	0.025*	
C9	0.5423 (2)	−0.0449 (7)	0.0884 (2)	0.0170 (7)	
C10	0.4704 (2)	0.0389 (8)	0.0388 (2)	0.0209 (7)	
H10A	0.4110	−0.0416	0.0480	0.025*	
C11	0.4848 (3)	0.2376 (9)	−0.0235 (2)	0.0266 (8)	
H11A	0.4350	0.2961	−0.0563	0.032*	
C12	0.5718 (3)	0.3529 (8)	−0.0386 (2)	0.0261 (8)	
H12A	0.5814	0.4879	−0.0820	0.031*	
C13	0.6447 (2)	0.2706 (8)	0.0099 (2)	0.0222 (7)	
H13A	0.7043	0.3476	−0.0006	0.027*	
C14	0.6299 (2)	0.0741 (7)	0.0741 (2)	0.0187 (7)	

### Atomic displacement parameters (Å^2^)

**Table:**

	*U*^11^	*U*^22^	*U*^33^	*U*^12^	*U*^13^	*U*^23^
S	0.0114 (3)	0.0242 (4)	0.0177 (4)	−0.0023 (3)	0.0028 (3)	−0.0015 (4)
O	0.0181 (12)	0.0470 (16)	0.0312 (15)	0.0089 (11)	0.0049 (11)	−0.0110 (13)
C1	0.0157 (14)	0.0217 (14)	0.0226 (19)	0.0006 (13)	0.0051 (14)	−0.0056 (14)
C2	0.0163 (15)	0.0174 (14)	0.0203 (17)	0.0046 (11)	0.0009 (13)	0.0016 (13)
C3	0.0113 (12)	0.0297 (15)	0.027 (2)	0.0020 (12)	0.0013 (15)	0.0002 (17)
C4	0.0167 (15)	0.0290 (18)	0.031 (2)	−0.0024 (14)	−0.0047 (15)	−0.0004 (16)
C5	0.0257 (17)	0.0275 (16)	0.0201 (17)	−0.0011 (15)	−0.0046 (15)	−0.0026 (15)
C6	0.0176 (14)	0.0205 (15)	0.0196 (15)	−0.0019 (13)	0.0039 (13)	0.0006 (14)
C7	0.0142 (13)	0.0141 (13)	0.020 (2)	0.0016 (11)	−0.0002 (12)	0.0000 (11)
C8	0.0200 (14)	0.0193 (16)	0.0228 (19)	−0.0038 (12)	−0.0014 (13)	−0.0017 (14)
C9	0.0205 (14)	0.0153 (15)	0.0153 (17)	−0.0009 (12)	−0.0005 (13)	−0.0048 (12)
C10	0.0189 (15)	0.0202 (16)	0.0235 (19)	−0.0007 (13)	−0.0016 (13)	−0.0050 (14)
C11	0.0299 (18)	0.0252 (17)	0.0247 (19)	0.0018 (14)	−0.0070 (15)	−0.0023 (16)
C12	0.0348 (18)	0.0255 (17)	0.0179 (16)	−0.0010 (16)	0.0040 (15)	0.0020 (14)
C13	0.0258 (16)	0.0216 (16)	0.0191 (16)	−0.0033 (13)	0.0075 (14)	−0.0040 (14)
C14	0.0179 (15)	0.0170 (14)	0.0211 (17)	0.0020 (12)	0.0048 (14)	−0.0043 (13)

### Geometric parameters (Å, °)

**Table:**

S—C7	1.760 (3)	C6—H6A	0.95
S—C8	1.798 (4)	C8—C9	1.485 (5)
O—C1	1.223 (4)	C8—H8A	0.99
C1—C14	1.500 (5)	C8—H8B	0.99
C1—C2	1.503 (5)	C9—C10	1.394 (5)
C2—C3	1.409 (4)	C9—C14	1.402 (5)
C2—C7	1.413 (4)	C10—C11	1.378 (6)
C3—C4	1.379 (5)	C10—H10A	0.95
C3—H3A	0.95	C11—C12	1.391 (5)
C4—C5	1.388 (5)	C11—H11A	0.95
C4—H4A	0.95	C12—C13	1.392 (5)
C5—C6	1.381 (5)	C12—H12A	0.95
C5—H5A	0.95	C13—C14	1.398 (5)
C6—C7	1.404 (4)	C13—H13A	0.95
			
C7—S—C8	104.17 (15)	C9—C8—H8A	109.1
O—C1—C14	119.5 (3)	S—C8—H8A	109.1
O—C1—C2	120.1 (3)	C9—C8—H8B	109.1
C14—C1—C2	119.5 (3)	S—C8—H8B	109.1
C3—C2—C7	118.0 (3)	H8A—C8—H8B	107.8
C3—C2—C1	114.0 (3)	C10—C9—C14	119.2 (3)
C7—C2—C1	127.8 (3)	C10—C9—C8	121.6 (3)
C4—C3—C2	122.2 (3)	C14—C9—C8	119.1 (3)
C4—C3—H3A	118.9	C11—C10—C9	120.5 (3)
C2—C3—H3A	118.9	C11—C10—H10A	119.7
C3—C4—C5	119.3 (3)	C9—C10—H10A	119.7
C3—C4—H4A	120.4	C10—C11—C12	120.4 (4)
C5—C4—H4A	120.4	C10—C11—H11A	119.8
C6—C5—C4	120.2 (3)	C12—C11—H11A	119.8
C6—C5—H5A	119.9	C11—C12—C13	120.0 (3)
C4—C5—H5A	119.9	C11—C12—H12A	120.0
C5—C6—C7	121.3 (3)	C13—C12—H12A	120.0
C5—C6—H6A	119.4	C12—C13—C14	119.7 (3)
C7—C6—H6A	119.4	C12—C13—H13A	120.1
C6—C7—C2	119.1 (3)	C14—C13—H13A	120.1
C6—C7—S	111.3 (2)	C13—C14—C9	120.1 (3)
C2—C7—S	129.6 (3)	C13—C14—C1	117.8 (3)
C9—C8—S	112.7 (2)	C9—C14—C1	122.2 (3)
			
O—C1—C2—C3	31.1 (4)	S—C8—C9—C10	−101.8 (3)
C14—C1—C2—C3	−137.8 (3)	S—C8—C9—C14	79.1 (3)
O—C1—C2—C7	−153.8 (3)	C14—C9—C10—C11	−0.3 (5)
C14—C1—C2—C7	37.3 (5)	C8—C9—C10—C11	−179.4 (3)
C7—C2—C3—C4	−2.1 (5)	C9—C10—C11—C12	1.2 (5)
C1—C2—C3—C4	173.4 (3)	C10—C11—C12—C13	−0.7 (6)
C2—C3—C4—C5	1.5 (5)	C11—C12—C13—C14	−0.6 (5)
C3—C4—C5—C6	−0.5 (5)	C12—C13—C14—C9	1.4 (5)
C4—C5—C6—C7	0.2 (5)	C12—C13—C14—C1	−177.9 (3)
C5—C6—C7—C2	−0.9 (5)	C10—C9—C14—C13	−1.0 (5)
C5—C6—C7—S	177.7 (3)	C8—C9—C14—C13	178.1 (3)
C3—C2—C7—C6	1.8 (4)	C10—C9—C14—C1	178.4 (3)
C1—C2—C7—C6	−173.1 (3)	C8—C9—C14—C1	−2.5 (5)
C3—C2—C7—S	−176.6 (2)	O—C1—C14—C13	−50.7 (4)
C1—C2—C7—S	8.5 (5)	C2—C1—C14—C13	118.2 (4)
C8—S—C7—C6	−172.2 (2)	O—C1—C14—C9	129.9 (4)
C8—S—C7—C2	6.3 (3)	C2—C1—C14—C9	−61.1 (4)
C7—S—C8—C9	−67.1 (3)		
